# Skin Surface Sebum Analysis by ESI-MS

**DOI:** 10.3390/biom14070790

**Published:** 2024-07-03

**Authors:** Madeline Isom, Heather Desaire

**Affiliations:** Department of Chemistry, University of Kansas, Lawrence, KS 66045, USA; madeline.isom@ku.edu

**Keywords:** lipids, lipidomics, sebum, skin, fingerprints, mass spectrometry, ESI-MS, machine learning, noninvasive sampling, biomarkers

## Abstract

The skin surface is an important sample source that the metabolomics community has only just begun to explore. Alterations in sebum, the lipid-rich mixture coating the skin surface, correlate with age, sex, ethnicity, diet, exercise, and disease state, making the skin surface an ideal sample source for future noninvasive biomarker exploration, disease diagnosis, and forensic investigation. The potential of sebum sampling has been realized primarily via electrospray ionization mass spectrometry (ESI-MS), an ideal approach to assess the skin surface lipidome. However, a better understanding of sebum collection and subsequent ESI-MS analysis is required before skin surface sampling can be implemented in routine analyses. Challenges include ambiguity in definitive lipid identification, inherent biological variability in sebum production, and methodological, technical variability in analyses. To overcome these obstacles, avoid common pitfalls, and achieve reproducible, robust outcomes, every portion of the workflow—from sample collection to data analysis—should be carefully considered with the specific application in mind. This review details current practices in sebum sampling, sample preparation, ESI-MS data acquisition, and data analysis, and it provides important considerations in acquiring meaningful lipidomic datasets from the skin surface. Forensic researchers investigating sebum as a means for suspect elimination in lieu of adequate fingerprint ridge detail or database matches, as well as clinical researchers interested in noninvasive biomarker exploration, disease diagnosis, and treatment monitoring, can use this review as a guide for developing methods of best-practice.

## 1. Introduction

### 1.1. Potential of Sebum Sampling

The skin surface is an intriguing sample source that allows for noninvasive collection methods and contains metabolites of great potential for both clinical and forensic applications. Most skin surface investigations focus on sampling from sebum, the lipid-rich residue coating almost the entirety of the human body [1]. These lipids are exceptionally complex and diverse. Importantly, sebum lipids may vary with age [2,3,4], sex [3,5], ethnicity [3], diet [6,7], and exercise [6]. This information could be particularly useful in narrowing down criminal suspects when no DNA matches exist in the database or when only a partial fingerprint is left at a crime scene (an amount inadequate for traditional identification) [3,7]. Sebum lipids also oxidize over time, generating products indicative of time-since-deposition [8,9], information that could eliminate fingerprints deposited at a crime scene prior to the crime taking place. Moreover, neurodegenerative diseases that currently lack definitive biomarkers are linked to changes in sebum production [10,11], suggesting sebum lipids may be analytes worthy of future biomarker exploration studies. Other illnesses, including diabetes [12], COVID-19 [13,14,15], cystic fibrosis [16,17], atopic dermatitis [5,18,19,20,21], and leprosy [22], are correlated with alterations in sebum lipids as well, indicating the potential for noninvasive disease diagnosis and monitoring. This painless sampling approach would be especially advantageous for vulnerable groups, such as children and the elderly, who are often excluded from traditional blood-based biomarker studies [23,24]. 

Despite the clear clinical and forensic potential for skin surface lipids, sebum sampling has yet to be implemented in routine applications. This lack of applications is likely due to the challenges associated with definitive lipid identification [25,26,27], the diverse range of sample collection, storage, and processing methods that directly impact the observed lipid profile [28,29], and the inherent variability in biological sebum production [28] that calls for sophisticated data processing and analysis [30]. Thus, significantly more research is needed to better understand and adequately address sebum lipid analysis and its challenges before non-invasive testing can be implemented into point-of-care practice and latent fingerprint collection can be a part of routine forensic investigations. 

### 1.2. The Skin Surface

**Figure 1:**
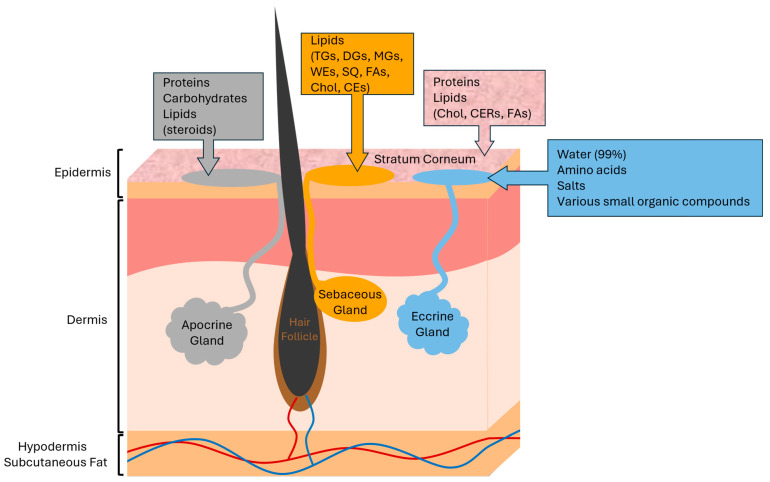
The origins of the predominant metabolites that are found on the skin surface. Abbreviations: CEs: cholesterol esters; CERs: ceramides; Chol: cholesterol; DGs: diglycerides; FAs: fatty acids; MGs: monoglycerides; SQ: squalene; TGs: triglycerides; WEs: wax esters.

Natural metabolites found on the skin surface have a mixture of both epidermal and dermal origins [25] as depicted in Figure 1. The epidermal stratum corneum (the outermost layer of the skin) [31] contains both lipids (mainly fatty acids (FAs), ceramides (CERs), and cholesterol (Chol)) and proteins [2,25,31,32], though molecules in this layer are often accessed with somewhat aggressive surface sampling means, such as tape stripping [25,33,34]. The dermis is responsible for the majority of readily available and easily extractable compounds located on the skin surface, and the molecules that come from this region reach the skin exterior through pores connecting to the apocrine, eccrine, and sebaceous glands [2,28]. The apocrine glands produce a miniscule portion of this mixture (namely small proteins, carbohydrates, and steroids), and they are only found in a few specific regions of the human body [35,36,37]. Eccrine glands can be found on the entirety of the human body, and they secrete a small amount of aqueous compounds, including salts, amino acids, and various small organic compounds, but they most predominantly secrete water (99%) [28,37]. This secretion of apocrine and eccrine glands is commonly referred to as “sweat” [30]. Sebaceous glands, which are present in all areas of the body except for the palms of the hands and soles of the feet [1,29], are thus responsible for the vast majority of easily extractable skin surface metabolites [2]. Sebaceous glands secrete metabolites in the form of sebum, which consists of various lipids, mainly glycerides, wax esters (WEs), squalene (SQ) and fatty acids (FAs), as well as smaller amounts of cholesterol (Chol) and cholesterol esters (CEs) [1,25,32]. 

Sebum-derived lipids are the primary focus of this review due to their majority presence and biological relevance, though it should be noted that, while miniscule in proportion, there are other interesting metabolites accessible via the skin. Additionally, it should be acknowledged that while this review refers to all skin surface lipids as “sebum lipids”, skin lipids are not always strictly sebaceous in nature, as this mixture could include epidermal origins (though this contribution is estimated to be roughly 30 times less than that of sebaceous origins) [2]. The exact skin lipid origins are difficult to identify and often depend on both the sampling method and anatomical collection region [4,34,38,39,40,41]. 

### 1.3. Sebaceous Glands

Sebaceous glands (SGs) are located at the shaft of hair follicles, and as such are a part of the pilosebaceous unit [1]. SG cells, known as sebocytes, produce and secrete the sebum lipid via a process known as holocrine secretion [1]. Sebum travels to the sebaceous duct, where it then takes about 2–3 weeks for the lipids to be deposited from the sebaceous duct onto the skin surface [32]. This process results in a readily extractable lipid mixture coating on the exterior of the skin. 

While many sebum lipids are synthesized within the sebaceous gland itself, others are brought in from the bloodstream [1,29]. Sebaceous glands produce receptors for the fatty acid transport protein (FATP) and low density lipoprotein (LDL), enabling the uptake of lipids from the bloodstream and their incorporation into sebum [29]. As a result, sebum lipids have been suggested as viable alternatives to blood-based biomarkers [30], and though only a few serum–sebum correlative studies have been conducted thus far, the preliminary results are promising [13,15,17]. 

Sebaceous glands are present on nearly every part of the human body, but the specific amounts can vary depending on the anatomical location [34,38,40,41]. The areas with the most SGs, including the face, back, and chest, tend to have the highest concentrations of sebum [29,34,38]. However, exact amounts of sebum lipids and the lipid class ratios vary among studies of the same sampling location, and this difference is likely due to variations in sampling protocols [29]. Common sampling protocols, and the corresponding effects on the observed lipid profiles, are further discussed in a subsequent section of this review.

### 1.4. Sebum Lipids and Contaminants

**Figure 2:**
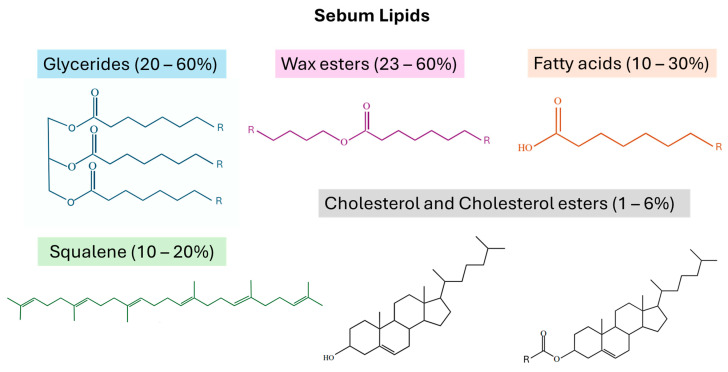
General structures and relative abundancies (%) of human sebum lipids.

Sebum lipids are tremendously diverse, but they include primarily glycerides (20–60%), wax esters (23–60%), squalene (10–20%), free fatty acids (10–30%), and cholesterol and cholesterol esters (1–6%) [1,2,25,29,32], as shown in Figure 2. Triglycerides (TGs), diglycerides (DGs), and monoglycerides (MGs), are lipids with a glycerol backbone and a number of fatty acid chains corresponding to their prefix [25]. Glycerides make up a large portion of sebum lipids, and they are often correlated with dietary changes [6]. Wax esters (WEs) consist of a fatty acid and fatty alcohol combined via esterification, and they constitute a significant amount of sebum lipids as well [2,28]. Squalene (SQ), a precursor to cholesterol, is more prominent in sebum than anywhere else in the body [1,29]. Cholesterol (Chol) has also been identified in sebum, but in trace amounts compared to its precursor, in a proportion unique to sebum [1,2]. Small amounts of cholesterol esters (CEs) are also present in sebum [2]. Fatty acids (FAs), which are hydrocarbon chains with a carboxylic acid end group, are another sebum lipid class commonly linked to diet changes [42]. Sapienic acid, the most highly accumulating fatty acid in sebum, is particularly unique to skin secretions, as it is strictly present only in sebum [2,25]. Ceramides (CERs), lipids with an amide bond connecting a fatty acid chain and a sphingoid base, are stratum corneum (SC) lipids that can also be found in sebum mixtures [31,32], when collection protocols involve SC extraction. Some of the most common lipids found in sebum have been reported in previous reviews [25,28].

While skin surface metabolites consist primarily of naturally secreted lipids, sebum mixtures can also contain contaminants. These contaminants can be indicative of an individual’s environment and behaviors. Sebum contaminants include chemicals from cosmetics, explosives, exogenous drugs, and air pollutants [7,42,43,44,45]. Microbes present on the surface of the skin can also be considered a type of contaminant, and these microbes have the potential to undergo chemical reactions with sebum lipids, in some cases altering the lipid properties [44].

### 1.5. ESI-MS Analysis of Sebum Lipids 

Several different analytical techniques have been used to analyze sebum lipids, including grease spot photometry [29,46], gas chromatography–mass spectrometry (GC-MS) [29], and matrix-assisted laser desorption ionization mass spectrometry (MALDI-MS) [33]; however, each has notable limitations. Grease spot photometry, often accomplished using a Sebumeter^®^, can only measure the amount of overall lipid content on the skin, lacking both specificity in analyte detection as well as absolute quantitation capabilities [46]. GC-MS is nonideal for the less volatile sebum lipids, including long-chain fatty acids and triglycerides, and often requires derivatization prior to analysis [26,47,48]. Conventional MALDI is nonideal for low molecular weight sebum lipids, such as fatty acids, due to matrix effects in the low mass range, though modified matrices may somewhat combat this issue [49].

Conversely, electrospray ionization mass spectrometry (ESI-MS) overcomes the shortcomings of other methods, and it is often the method of choice for lipidomic analysis [26,33]. ESI-MS is a soft ionization technique in which solvent droplets are ionized via a high voltage needle, transferred to the gas phase due to Coulombic repulsion, and delivered to the mass spectrometer via an electric field. ESI-MS allows for multi-analyte profiling with high sensitivity, specificity, and efficiency that is ideal for the complex mixture of sebum, and the technique is also well-matched with the lipidomic mass range (approximately 100–2000 *m*/*z*). For added specificity, ESI-MS can be paired with pre-acquisition separation techniques as well as further tandem MS experiments. Considering the hydrophobic nature of the lipid analytes in sebum [29], ESI is a particularly ideal ionization choice for this application, as hydrophobic molecules (lipids) tend to have a higher ionization efficiency compared to hydrophilic compounds [50,51]. However, ESI-MS does have a few notable drawbacks. The high salt content in natural skin secretions can be problematic for ESI-MS, as salts can compete for analyte charge, decrease analyte volatility, and clog the ionization source, all of which can depress the analyte signal [51]. Ion suppression from more concentrated analytes is also a concern in ESI-MS, as well as carryover effects from consecutive injections and instrument drift that can skew comparative results [24,27,52,53,54]. These challenges can be addressed via intentional sample preparation and proper data acquisition and processing methods, which are discussed in this review.

**Figure 3:**
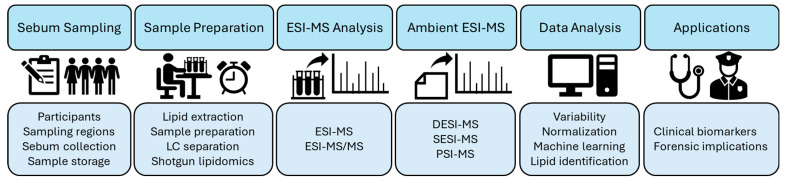
Overview of topics for ESI-MS sebum analysis review. Abbreviations: DESI: desorption electrospray ionization; ESI-MS: electrospray ionization mass spectrometry; LC: liquid chromatography; PSI: paper spray ionization; SESI: secondary electrospray ionization.

This review describes current methods for analyzing the lipid profiles of skin surface sebum lipids via ESI-MS, including a summary of current practices in the sample collection, sample preparation, data acquisition, and data analysis of sebum lipids (overview depicted in Figure 3). In addition, we highlight important considerations in method development that will allow researchers to overcome obstacles and avoid common pitfalls, as well as achieve results optimized to their specific application of interest. Skin surface sebum analysis via ESI-MS is sure to propel the field of metabolomics forward and has the potential to one day change the way we consider both clinical evaluations and forensic investigations entirely. This review outlines the efforts currently put forth to achieve this incredible, yet attainable, goal. 

## 2. Sebum Sampling from the Skin

### 2.1. Overview of Skin Surface Sampling Techniques

The sebum sampling technique directly impacts the observed lipid profile [29], and therefore is a crucial aspect to consider in the sebum analysis workflow. Participant demographics, as well as the participant regimen prior to sampling, may affect the reproducibility and robustness of the results [28,29]. Temperature and humidity during collection can impact the quantities of various sebum lipids obtained, and likewise, profiles have been shown to vary with season [29,40]. Anatomical regions of collection differentially impact the resulting analyte distribution and concentration [4,34,38,39,40,41], so samples collected from different regions of the body will not necessarily result in comparable lipid profiles. The substrate choice, as well as the specific collection method, will determine the depth of skin penetration and degree of extraction, thus impacting the resulting lipid profile [4,42]. Since the sampling method will directly influence both the observed lipid profile and the overall utility of the analysis, it is critical that each aspect of the sampling protocol be chosen intentionally for the desired application. 

### 2.2. Participant Selection and Regimen Prior to Sampling

Most sebum analyses rely on samples collected from multiple individuals, and it is important that these individuals are chosen in a way that addresses the unique goal of the study. Researchers have shown that a participant’s sex [3,5], ethnicity [3], age [2,3,4], diet [6,7], and exercise [6] can each impact the lipidomic profile observed. Therefore, when the goal of sebum analysis is to be applicable to the entire population, the chosen participants should be representative of the entire population, especially with respect to the specific differences known to impact sebum lipids. Though some sebum biomarker studies contain equal representation of males and females, most of the studies include only participants from a narrow age range or single racial group. While the results from such experiments are useful for the specific demographic studied, there may be different biomarkers that would be more optimal for the groups that were not included in the experiment; this finding, that nondiverse sample sets lead to non-optimal biomarkers for excluded groups, has been demonstrated in biomarker studies involving other biofluids [55]. It is important, therefore, to consider the inherent participant-based variables affecting sebum secretions when choosing a participant pool. For the most robust results, the participant pool should be diverse. 

Participant regimen prior to skin surface sampling can also affect the observed lipid profile, and as a result, many studies seek to standardize this variable. Some regulate the use of cosmetics and perfumes prior to sampling [5,56,57,58,59,60], while others screen for medication use, alcohol intake, and smoking habits [5,20,61]. Several studies employ a pre-sampling acclimation step, in which the participant is required to sit in the room of sample collection for 15–30 min prior to sampling [12,40,56,61,62,63]. In many methods, participants are instructed to wash their hands before sampling, and some protocols even standardized the use of soap or lack thereof [12,40,56,61,62,63,64,65]. Others require the sampling site be cleansed by the researcher with solvents like isopropanol (IPA) and ethanol [5,19,39,40,66,67,68,69]. While all of these efforts may decrease intragroup variability, thus enhancing intergroup distinctions in reference to the variable of interest, they also risk producing results that cannot be directly compared to other studies. Additionally, it is impossible to control the participant regimen in the case of crime-scene sebum deposits. For these reasons, a data normalization step is critical. Normalization strategies are discussed in a later section of this review. 

### 2.3. Anatomical Collection Regions 

The region of the body from which sebum is collected can have a measurable impact on the resulting lipid profile, and as such, this variable should be considered with respect to the goal of the study. In different regions of the body, not only are different amounts of sebum secreted, but also different ratios of analytes [4,34,38,39,40]. Consequently, the region of collection should reflect the specific aim of the work. For example, if the goal is to identify biomarkers of a particular disease, and the disease primarily affects a population known to produce lower amounts of lipids, then regions of maximum sebum production should be chosen, like the face, back, and chest [29,34,38]. Similarly, if a specific lipid class is known to correlate with the disease of interest, then anatomical regions that produce higher concentrations of the target analytes should be chosen. More research needs to be performed to fully understand which lipids are most abundant at which collection sites. Likewise, if the most often identified skin-print at a crime scene is a finger or handprint, then forensic-driven studies should sample from these locations to achieve the most applicable results. Since the anatomical sampling region can affect the lipid profile, it should be chosen with the long-term goal of the study in mind. 

While the most common sebum samples for ESI-MS are collected directly from the forehead, forearm, cheek, back, and fingertips (Table 1), some studies also incorporate “grooming” techniques [9,24,42]. Grooming is a method in which the participants are asked to briefly rub or touch their finger to a region of their body that is higher in sebum production, most often the face, prior to depositing a latent fingerprint. This approach aims to enhance the detection of analytes that exist in very low abundance on the natural, un-groomed fingerprints. While grooming techniques can certainly be useful in increasing the overall MS signal of low-abundance lipids, it is important to consider that the grooming region can impact the lipid profile observed and may introduce unnecessary variability into the collection method. Studies show that different regions of the face can produce different lipid profiles [38,40], suggesting that when grooming techniques are employed, they should be confined to a single location for optimal reproducibility.

### 2.4. Collection Methods for Skin Surface Lipids 

The collection substrate and sampling protocol will affect both the amount and type of lipids collected [28,29,42]. This fact is especially important to consider in forensic-driven applications, in which the initial crime-scene sebum deposition cannot be controlled. For this reason, several studies have explored the lipid profile resulting from various substrates of deposition [28,29,42]. However, in clinical applications, where sampling can be directed by personnel, the sampling substrate and protocol can be controlled, and therefore optimized, for reproducible collection. The most commonly used materials for skin surface lipid collection prior to ESI-MS include tape strips, silica plates, glass slides/vials, cotton gauze/swabs, cyanoacrylate resin, and aluminum foil (shown in Table 1).

Tape stripping and cyanoacrylate resin are almost exclusively used for ceramide analysis, since both penetrate the stratum corneum, extracting ceramides from this layer as well as the sebum lipids above. The most common tape strips used in ESI-MS sebum studies are Sebutape^®^ and D-Squame^®^. While some studies utilize a single tape strip worn by the participant for a matter of minutes [61,62,64], most incorporate more aggressive protocols. Several protocols repeat tape stripping in the same location with multiple strips to remove contaminants and reach deep into the stratum corneum layer [4,18,20,21,40,59,60,65,68,69,70,71]. In other cases, the tape strips are left on the participants for up to 1 h [5,19]. A cyanoacrylate resin, similar to super glue, has also been employed for SC ceramide extraction in lieu of tape stripping [39,72,73]. While tape strips and cyanoacrylate resin are often not the most ideal substrates in terms of comfort and throughput, they are the substrates of choice for sufficient ceramide class coverage, and these lipids have been shown to correlate with various skin diseases [18,19,20,21,61,74,75]. 

In cases where ceramides are not the lipid class of primary interest, ESI-MS-based sebum analyses utilize other collection methods. While these alternative protocols do not disrupt the SC, many ceramides can nonetheless be present in the extracted sebum mixture [10,14,16,63]. These methods (shown in Table 1) include depositing latent fingertips onto aluminum foil pieces [9,24] or into scintillation vials [8], pressing a glass slide to the skin surface [3,17,63], pressing a silica plate to the skin surface [13,16,22], swabbing the collection site with a cotton swab [7,11,58] or gauze [10,14], and sampling with a stainless steel rod [38]. All of these collection methods take minimal time (approximately one minute per sample collected) and require no discomfort for the sample donor. However, they also lack the depth capabilities needed to extract abundant lipids from the SC, so the majority of these lipids are sebaceous in nature.

In addition to controlling the sampling substrate, many protocols also standardize the sampling environment. Temperature, humidity, and collection time are often controlled variables during sebum collection. Since these variables can impact the lipid profile [29,40], the most reproducible sampling method will consider all of these. However, for robust implementation of skin surface sampling across institutions, either in future point-of-care practice or investigative forensic work, the sampling environment will likely vary and, as such, so may the resulting lipid profile. A post-acquisition normalization method could be useful in minimizing this inevitable variability of sampling environment. 

### 2.5. Sample Storage

Sample storage is an important consideration in the sebum analysis workflow as lipid deposits have been shown to oxidize over time [9]. Squalene, glycerides, cholesterol, and fatty acids tend to decrease over time, and small oxidative products emerge simultaneously as a result [8,9,28,63,76]. While lipid oxidation is useful in forensic applications of time-since-deposition [8,9], it can also be a detriment when the goal of the study is to compare disease vs. control groups collected from different people at different times. To overcome this issue, dark storage conditions, reduced air circulation, and low temperatures may slow down lipid degradation [9] and allow for comparable samples despite different collection days. However, the extent to which different storage conditions affect the sebum lipid profile has yet to be thoroughly explored.

## 3. Sample Preparation Prior to ESI-MS

### 3.1. Lipid Extraction Methods 

Liquid–liquid extraction is the method of choice for sebum lipid extraction; however, no single method or solvent system is equally ideal for all lipid classes [26,29]. As such, the extraction conditions should be chosen with the target lipid classes in mind. The most common method of sebum lipid extraction prior to ESI-MS is the Bligh and Dyer method [80]. In this protocol, lipid extraction is carried out with an initial 1:2:0.8 ratio of chloroform: methanol (MeOH): water (*v*/*v*). After mixing for 2 min, an additional aliquot of chloroform is added to achieve a 2:2:0.8 ratio, 30 s of mixing occurs, and then water is added to a final ratio of 2:2:1.8, followed by 30 s of mixing. After a rapid filtration step, the filtrate undergoes liquid–liquid extraction in which the methanol portion is removed, and the chloroform layer constitutes the resulting lipid extract. The entire extraction takes approximately 10 min. In several cases, variations of the Bligh and Dyer method are used for sebum analysis [12,20,61,62,64,71,77]. For example, acetone (ACE) is frequently incorporated into the method in analyses aimed at multiple lipid classes with diverse polarities (FAs, TGs, DGs, CERs) [12,61,64]. 

Initial extraction with ethanol (EtOH) followed by liquid–liquid extraction using ethyl acetate has proven capable of extracting a wide range of lipid classes (TGs, DGs, MGs, WEs, FAs, CEs, and SQ) as well [66,67]. Likewise, MeOH/water extraction is also effective in multi-class analysis (ceramides, FAs, DGs, TGs) [13,16], followed by further separation via re-extraction steps with chloroform in ceramide-focused studies [19,20,21,65,74]. When there is a single lipid class of interest, however, optimal solvents are often chosen with the specific lipid class’s hydrophobicity in mind. Since more nonpolar solvents will extract more nonpolar analytes, like triglycerides, and more polar solvents will extract more polar analytes, like fatty acids, optimal solvents can be chosen based on the target analytes’ polarity. EtOH/water extraction has been useful in FA analysis [7], chloroform/water and dichloromethane/MeOH in TG analysis [9,24], and hexane/EtOH in ceramide analysis [39,72,73]. 

While liquid–liquid extraction is a common method of sebum sample purification, other techniques, including solid-phase extraction (SPE) are often incorporated into sebum sample prep, primarily in the case of ceramide analysis [18,40,41,60,69,70]. SPE is used in ceramide analysis, especially prior to positive ion mode ESI-MS, as a means for removing unwanted contaminants from the tape strip and skin that otherwise interfere with lipid analysis [69]. SPE is most useful in targeted studies where further fractionation is needed [26]. 

### 3.2. Sample Preparation Prior to ESI-MS

As in the original Bligh and Dyer method, most sebum lipid extractions separate the aqueous from the organic components in the lipid mixture, discarding the aqueous layer prior to analysis. Removing the aqueous component purifies the mixture of naturally secreted salts that would otherwise be detrimental to ESI-MS, and it further concentrates the analytes. Since sebum metabolites are in relatively low abundance, many extraction protocols are followed by additional concentrating steps. Concentrating usually entails nitrogen evaporation and reconstitution of the lipid droplet in a smaller volume (µL scale) to enhance analyte concentration and thus MS detection. Final lipid extracts are then reconstituted in mixtures of two to three solvents of varying polarities, most often IPA, MeOH, dimethyl ether (DME), ACE, EtOH, chloroform, or acetonitrile (ACN) (Table 1). Most protocols also include the addition of an MS-friendly additive, such as formic acid or ammonium acetate, to facilitate efficient separation and ionization for optimal MS detection.

When the goal of the study is quantitative in nature, and in instances of extraction recovery evaluation, protocols often incorporate the addition of lipid-based internal standards. Due to the expansive number of diverse lipids, standards are usually chosen to represent each lipid class. Commonly used standards are deuterated forms of cholesterol [66], cholesterol sulfate [34], ceramides [34,60,65], triglycerides [66], fatty acids [34,71], and nondeuterated non-endogenous lipids [77] or lipids with endogenously low abundancies [75]. In quantitative studies that involve tape stripping, researchers also commonly weigh the tape strips before and after collection [34,66,67]. 

### 3.3. Liquid Chromatography Separation

Most ESI-MS sebum analyses use liquid-chromatography (LC) separation prior to MS injection to maximize analyte specificity and evade ion suppression. Both reversed-phase and normal-phase LC are used, and the choice is dependent on the target analytes. For ceramide analysis, both normal-phase silica columns [18,40,41,59,70] and reversed-phase C-18 [12,39,65,69], C8 [60,75], and amide [20,21,74] columns have been chosen. For other lipid classes, reversed-phase LC, either with a C18 [7,9,10,14,15,19,61,62,63] or C8 column [5,34,66,67], is most common. Reversed-phase LC is common for lipid analyses because the alternative, normal-phase LC, separates lipids by their head group polarity, resulting in the coelution of lipids from the same class and consequential inability to separate isomers [33]. However, since reversed-phase LC separates lipids based on their nonpolar components, which vary between lipids of the same class, intra-class lipid separation is possible with reversed-phase LC [26,33]. Additionally, an ion-pairing agent, such as formic acid, ammonium acetate, or ammonium formate, is often added to the mobile phase to adjust the pH and facilitate eventual analyte ionization.

### 3.4. Shotgun Lipidomics

Not all sebum analyses utilize LC prior to ESI-MS. In some instances, flow injection or “shotgun” methods are chosen as a means for achieving maximum efficiency; example studies are shown in Table 1. While fewer analytes are observed overall in this approach, the resulting lipidomic profile can be sufficient for certain analysis goals, such as the distinction between sample types that are differentiable based only on the abundant lipids [13,16,22,24]. In lieu of upfront separation, direct injection increases throughput, and this feature is especially advantageous when machine learning is leveraged during data analysis, since algorithms often benefit substantially from datasets with more samples. The single retention time window also bypasses any further data processing steps that would otherwise be necessary for retention time alignment, another feature that facilitates workflows incorporating machine learning. A simple approach, shotgun lipidomics, is gaining momentum, especially in applications focused on high throughput. However, for a more wholistic picture of the lipidomic profile on the skin surface, shotgun lipidomics falls short. In traditional shotgun lipidomics, many lipids elute at once, causing issues of ion suppression and mixed MS/MS spectra from isomers [26,52]. For this reason, shotgun lipidomics is best applied when deep coverage is not the focus of the study and efficiency is. 

## 4. ESI-MS and ESI-MS/MS of Sebum Lipids

### 4.1. ESI-MS

ESI-MS is a soft ionization technique that allows for accurate and sensitive lipid ion detection [33,51]. ESI-MS analysis of sebaceous lipids is performed in both positive and negative ion modes, and in both cases, these lipids almost exclusively form singly charged ions. In positive ion mode, [M+H]^+^ and [M+H-H_2_O]^+^ ions are common, as well as sodium, ammonium, or acetate adducts [4,9,11,22,65,66,70]. In negative ion mode, [M-H]^−^ and [M+Cl]^−^ (in the case of ceramides) are the most commonly reported sebum lipid ions [16,69,72,77]. Positive ion mode is most often used in analyses aimed at identifying lipids from a wide variety of classes, including squalene, glycerides, fatty acids, ceramides, and wax esters. Negative ion mode is often chosen when fatty acids are the primary lipid class of interest [8,17,34,56,57,67], as the specificity and sensitivity for fatty acids is higher compared to positive ion mode (up to 10-fold difference) [67]. However, ceramides and diglycerides have also been analyzed in negative ion mode [16,34,72,73,77]. The ionization mode can thus be chosen optimally with the target lipid classes in mind. The mass range that encompasses all sebum lipids, regardless of ionization polarity, is within approximately 100 to 2000 *m*/*z*. 

### 4.2. ESI-MS/MS

For accurate lipid class identification, tandem MS is required. In the case of sebum lipidomics, tandem MS is accomplished with multiple reaction monitoring (MRM) [38,60,71,75], selected ion monitoring (SIM) [41], a neutral loss scan (NLS) [67], or a product ion (PI) scan [34,67,70]. Diagnostic ions for each lipid class correspond to the lipid head groups associated with each class. Glycerides tend to fragment at the glycerol backbone, and the fragmentation patterns are indicative of the fatty acid chains [4,9,11]. Wax esters tend to fragment at the ester linkage, producing a fragment similar to that of a fatty acid precursor ion [67]. Cholesterol and cholesterol esters notably form the diagnostic ion *m*/*z* 369 (C_27_H_45_), indicating a break between the fatty acyl chain and cholesterol head group [38,67,81]. Ceramides fragment into the sphingosine base and fatty acid components, from which their structure and corresponding subclass can be identified [18,39,70]. Though ceramides can be detected in both positive and negative ion modes, positive ion mode is preferred for tandem MS experiments. This mode is preferable because in positive mode, [M+H]^+^ ions are the most abundant ceramide precursor ions compared to adducts, but in negative ion mode, [M-H]^−^ ions are often less abundant than [M+Cl]^−^ adducts. Richer fragmentation patterns are, therefore, observed in positive ion mode, so it is the mode of choice for ceramide profiling [69]. 

Though tandem MS is very powerful in narrowing down the structural identity of the lipid, it is not always capable of a definitive assignment. Several lipids are isomeric and isobaric; therefore, the isolated precursor *m*/*z* value can produce a complex fragmentation spectrum [11,67]. In the case of triglycerides, the possible presence of multiple species with the same nominal mass can make assigning the exact acyl chain lengths and double-bond placements extremely difficult or impossible. While LC-ESI-MS methods have shown isobaric TG species may coelute, resulting in nonsymmetric peak shapes [67], more recent efforts show that the use of ion mobility for collision cross-section (CCS) mapping can achieve isobaric TG discrimination [11]. 

## 5. Ambient ESI-MS Approaches to Sebum Analysis

### 5.1. Overview of Ambient ESI-MS

Recently, ambient ESI-MS techniques that enable the ionization of molecules from a solid surface, rather than from solution, have been growing in popularity. These techniques are advantageous because they require little to no sample preparation, thus allowing for optimal workflow efficiency. In addition, since the substrate or skin surface is analyzed directly, lipid extraction is not necessary, and therefore concerns of both analyte loss during liquid extraction and analyte bias from solvent polarity are avoided [29]. Desorption electrospray ionization (DESI), secondary electrospray ionization (SESI), and paper spray ionization (PSI) combine conventional ESI-MS analysis with imaging capabilities, real-time analysis, and extremely high-throughput potential. This combination of features makes ambient ESI-MS methods especially suitable for acquiring large sets of samples, and therefore large datasets, which is beneficial for machine learning algorithms, when the goal is to decipher which sets of lipids distinguish one group from another (i.e., diseases vs. control). Since machine learning on large datasets will be a critical predecessor to future sebum-based disease diagnosis, ambient ESI-MS techniques represent a steady foot forward in this regard.

### 5.2. DESI-MS

DESI [82] is a technique that allows for ambient analysis of solid samples (the substrate of collection or the skin surface itself) using electrospray ionization [78,83,84,85]. In DESI, ESI microdroplets desorb the analyte ions from the sample, and then the gas-phase ions are directed into the MS [82]. DESI is especially useful in discerning the distribution of lipids on the skin surface or imprint [33]. Lipid ions of interest can be chosen and mapped across the entirety of the sample, depicting the analyte’s abundancy distribution, though the imaging resolution tends to be lower than that of MALDI-MSI [33,84]. Contrary to MALDI imaging, however, DESI requires no matrix, and thus matrix effects are evaded [82]. 

DESI has been used to analyze the way the lipid distribution changes with consecutive tape stripping in the same sample site, as the layers of the stratum corneum are further removed [83]. It has also been used to monitor transdermal drug administration [83]. In another study, researchers used DESI to identify potential biomarkers of cystic fibrosis, with a 2 min sampling/acquisition process that required wiping a glass slide on the forehead of the donors (compared to the 3 h current diagnosis of CF using sweat testing of Cl^−^ ions) [17]. Researchers have also used thermal DESI to explore the distribution of squalene throughout the entirety of the human body by sampling from 1357 sample sites of a single donor, a process that took just 15 s per measurement [38]. Another group used DESI to understand the lipid distribution in fingerprints for forensic applications; they were able to distinguish between donors of different ethnicity, age, and gender using machine learning methods [3]. Similarly, others have used DESI to analyze variations in fingerprints when deposited on different types of surfaces, work that will be important to consider if this technique is to be implemented in crime-scene investigations [42].

However, like every method, DESI-MS has a few notable drawbacks. While DESI microextraction allows for some pre-acquisition separation [84], it fails to enable the same level of analyte separation achieved with LC techniques, especially in the case of isobaric species [86]. Consequently, biological matrix effects, which vary among analytes, are a common pitfall of ambient MS methods like DESI [87]. Moreover, since internal standards are not easily incorporated into solid samples, adequate quantitation with this method can be challenging, a problem that is further hindered by insufficient peak stability from surface charging [86,87]. In general, sufficient specificity and accurate quantitation are difficult to achieve with DESI-MS, and improvements in these areas would enable researchers to better leverage the high-throughput potential of the technique.

### 5.3. SESI-MS

SESI [88,89] is considered to be a softer ionization technique than conventional ESI, in which volatile metabolites from the sample source directly interact with the electrospray plume, undergo ionization, and are analyzed via MS [90]. SESI has been used to investigate the O_3_ reactions of skin lipids via rapid, real-time measurements [76]. In this experiment, an individual placed their entire hand inside a sealed bag where ozone was introduced, and ozonolysis products were realized [76]. In another study, SESI was used in combination with thermal desorption (TD) for skin patch sample analysis to be used in future diagnostics [57]. SESI is a sensitive method that allows for efficient, real-time MS data acquisition; however, like DESI, the method lacks the depth of coverage obtained by LC-MS methods, and unwanted interference from external compounds (ex. fragrant hygiene items) is common [91].

### 5.4. PSI-MS

PSI is a variant of ESI in which a solid or liquid sample is extracted from filter paper with a sharp point under ambient conditions [92]. The filter paper is placed near the MS opening, a high voltage is applied behind the paper, and solvent is applied onto the sample. Molecules are extracted by the solvent and then ionized via ESI from the electric field [79]. Paired with ion-mobility separation, PSI has been used to analyze differences in the sebum lipids of Parkinson’s disease patients vs. healthy controls [11]. A variant of PSI, zero-volt paper spray ionization (zvPSI) [93], has also been used in skin lipid analysis [79]. Unlike PSI, this method does not require gas or high voltage [79]; however, it tends to have a somewhat lower ionization efficiency compared to PSI [83]. Zero-volt paper spray ionization is capable of analyzing liquid or semi-solid samples by extracting them with solvent from a filter paper in ambient conditions before ionizing via solvent-assisted inlet ionization [83], and it has been used to rapidly analyze the lipids present on the face [79]. PSI is an alternative to traditional ESI-MS that has been useful in multiple sebum analyses; however, the rapid consumption of the ionization solvent greatly limits the time available for data acquisition—a pitfall many researchers have attempted to overcome with various PSI modifications [94].

## 6. Data Analysis of Sebum Lipids

### 6.1. Overview of Data Analysis Approaches

Analyzing the MS data acquired from sebum sampling can be a difficult undertaking, and the approach should be considered carefully to avoid bias and achieve useful, reproducible results. The types of variability inherent to sebum analysis include both biological and instrumental variations, and these are critical to consider when comparing sebum samples. Different normalization strategies are used to combat this variability, including normalization to an internal standard [8,9,34,66], quantile normalization [13], Progenesis [11,12,14,64], normalization to the sum of peaks in the lipid class [63], normalization to the total sum of peak intensities [3,58], normalization to the sample weight [34], total peak area normalization [10,67], and normalization to the total protein content [20,60,75]. After data normalization, the classification of groups with respect to a specific variable of interest (for example, disease state) is achieved via either machine learning methods or classical statistical analyses. The *m*/*z* values that are most important in distinguishing the groups of interest could then be characterized (if not yet performed) and considered potential biomarkers. Lipid identities are often deduced using tandem MS fragmentation data in comparison with those found in lipid databases such as METLIN [95], MassBank [96], Human Metabolome Database (HMDB) [97], LipidHunter [98], LipidXplorer [99], LipidSearch (Thermo Fisher Scientific), and LIPID MAPS [100]. 

### 6.2. Types of Variability

Sebaceous gland production varies between individuals [24,28], and even within the same individual throughout a single day [6]. Sebum variations between different individuals are linked to diet [6,7], age [2,3,4], ethnicity [3], and sex [3,5]. For example, free fatty acids are less abundant in children’s fingerprints than in adults’ [28], and the lipids in children’s fingerprints vary more than those in adults, which may be a result of hormonal changes related to puberty [28,29]. Other age-linked hormonal changes, including menopause, are correlated with changes in ceramide levels [60]. In some adult sebum studies, males and females are shown to secrete different lipids related to their sex [3,5]. Specifically, one study has indicated that males and females have differences in the levels of fatty acids and ceramides [5]. However, other studies have contradicted these results [4,6,28]. In reference to diet, specific sebum differences related to vegetarianism have been observed, with vegetarians producing higher concentrations of saturated triglycerides than non-vegetarians [6]. In reference to exercise, males are reported to secrete less saturated triglycerides when their daily routine involves regularly exercising [6]. Sebum production has also been shown to correlate with temperature changes [28], and therefore lipid profiles could vary depending on the season and geographical location of collection [29,40,75]. Higher temperatures are associated particularly with the increased degradation of sebum esters [28], and specific seasonal correlations are evident in skin ceramides [40,75]. 

Despite the high potential for lipid sampling from sebum to provide diagnostic information on disease states, researchers who wish to apply these approaches may need to pay special attention to the numerous factors that can cause variations in sebaceous gland production. This point was demonstrated by Spick et al. in a study comparing sebum samples from COVID-19 patients to healthy controls, in which subgroups of participants with common medications or health conditions showed a better classification of disease vs. control than was observed using all samples [14]. Current methods to account for the inevitable biological variability in sebaceous gland secretion, and its subsequent effect on biomarker exploration, are discussed in the following section.

Aside from the biological variability in sebum samples, instrumental variability is also important to consider in the sebum analysis workflow. The instrumental drift, an inherent issue in mass spectrometry experiments, introduces unwanted batch effects that can make data analysis more challenging [53,54]. For example, in MS experiments with several consecutive sample injections, it is common for samples in the first half of the run to be much more similar to one another than to samples injected in the second half of the run. This variability is due to MS signal drift over time. In very long injection series, where many samples are required for analysis, these batch effects can become even more apparent, sometimes even more so than the biological variable of study [24]. As a result, when samples are injected in order with respect to the variable of interest (for example, when all of the control samples are injected first, and then all of the disease samples are injected in the last half of the series), there may be an artificial distinction between the two groups [54]. It is then impossible to tell if this differentiation between disease and control groups is truly a result of disease state or simply a result of injection time as introduced by MS signal drift. As has been previously reported, randomized sample injection is key to obtaining authentic, unbiased results [24,53]. Additionally, in cases where samples must be injected on multiple different days, it is critical that a normalization strategy be employed to account for these inevitable batch effects.

Aside from instrumental variability in signal drift, other technical variations to consider are carryover from multiple sample injections and column degradation [53]. Quality control samples and blank injections can help to assess these issues and maintain reproducible, reliable results. Sufficient washing between each sample, using solvents with varying polarities, can also aid in ensuring technical variability does not interfere with the analysis.

### 6.3. Normalization Strategies 

The inherent variability (both biological and systematic) of sebum lipid analysis requires adequate data processing strategies to achieve meaningful datasets. This fact is especially true when comparing samples that were collected from different individuals and analyzed on different days. The reproducibility of the data, as well as the accuracy of the classification, is often a result of the strength of the normalization strategy chosen (and good experimental design overall). For example, one study collected sebum samples from multiple different hospitals in different geographical regions, and with the use of normalization, was still able to distinguish between Parkinson’s disease and healthy control samples, despite the inevitable variability in sampling [10]. 

In ESI-MS sebum analysis, normalization is often accomplished by scaling the MS intensity values to a common factor, such as the intensity of the internal standard [8,9,34,66], sum intensity of a specific lipid class [63], total sum intensity of all peaks [3,24,58], total peak area sum [10,67], total protein content [20,60,75], or sample weight [34]. In the majority of these normalization strategies, the overall distribution of the lipid profile is unchanged [27]. However, other strategies are also possible, like quantile normalization, which has also been used in ESI-MS sebum analysis [13]. This method aims to make the overall lipid distributions the same between samples, thus changing the relative intensity distribution within each sample [27,101]. Another common normalization method for skin lipids, Progenesis normalization (via Progenesis software, Waters Corporation, Milford, MA, USA) [11,12,14,64], normalizes samples to a reference sample [101]. The best normalization strategy, however, is specific to the lipidomic workflow and application, and several different approaches should be considered. For example, one group found that normalizing to TG 48:0 was well suited for fingerprint aging analysis [9]. Choosing a normalization strategy with regards to overcoming systematic variability has been outlined previously by Chua et al. [102]. 

### 6.4. Machine Learning Methods

Several studies that implement machine learning into the workflow do so to identify potential biomarkers in sebum with regards to disease state or donor demographics. In these studies, both supervised and unsupervised classification strategies have been implemented (see Table 1). Supervised models include various decision-tree based strategies, including random forest (RF), gradient boosting tree ensemble (GDBT), and extreme gradient boosting (XGBoost), along with many other methods, such as partial least squares-discriminant analysis (PLS-DA), orthogonal partial least squares discriminant analysis (OPLS-DA), support vector machine (SVM), linear and logistic regressions, and deep learning. A commonly used unsupervised model is principal component analysis (PCA). Supervised models are particularly useful for identifying when there are a group of potential biomarkers important in distinguishing between groups, and the variable of discrimination is known, while unsupervised models allow for researchers to identify when global distinctions exist within the dataset. 

PCA, an unsupervised visualization method, is a dimensional-reduction technique that plots the data points on a newly established axis, which are derived in a way that enables the visualization of maximum variability between data points [103]. In this method, a plot is produced in which points that are nearby one another have more in common than points that are farther away from one another. The researcher can then identify apparent variables discriminating between sample clusters and determine which experimental conditions contribute most to the global variability, thereby offering insights about how the results might be improved upon. For example, PCA is useful for choosing an effective normalization method [102].

Supervised techniques are applicable when the variable of interest is known, and the samples belonging to the control and experimental group are also known. PLS, PLSD, PLS-DA, and OPLS-DA are similar to PCA in the sense that these utilize dimension-reduction, but different in the sense that they use the known group-identification of each sample to cultivate predictor and response variables [27]. These methods are easy to implement and are frequently used on MS data, but they perform best with collinear datasets [104]. Gradient boosted trees and random forest are other types of supervised models that work by decision-tree approaches in which the features most useful in discriminating between known sample groups are identified and then used to construct a series of branch points for classification of unknown samples [105]. XGBoost is a particularly powerful adaptation of this general approach. The support vector machine (SVM) utilizes the kernel function and Radial Basis Function to determine a boundary to be used in group classification; however, this model can be prone to overfitting bias [104]. Linear and logistic regression models are also used for supervised prediction models.

Since most models cannot process datasets with missing values, it is common for researchers to fill in these missing values prior to data visualization or classification [104]. This step, called imputation, can be performed in a variety of ways; one common strategy is to use a Bayesian model [11]. An alternative way to deal with this problem is to use a classifier, like XGBoost or AC.2021, which is able to tolerate missing data [106]. Another strategy to consider, which both decreases the overall number of missing values and reduces the dimensionality of the data, is to sum peaks within a defined *m*/*z* range, rather than using every peak as a feature in the model. This process is streamlined via the LevR algorithm [107], and it has been used in subsequent sebum ESI-MS analysis [24].

Model sensitivity and specificity are often assessed by generating receiver operator characteristic (ROC) curves [10,12,13,21,24]. ROC curves are constructed as the true positive fraction vs. false negative fraction, and the area under the curve (AUC) is calculated as a means of evaluating model sensitivity and specificity [108]. An AUC close to 1 represents a classification that can be achieved with perfect accuracy—each sample is correctly identified as belonging to its corresponding group. Alternatively, an AUC close to 0.5 represents a classification in which only half of the samples are correctly identified as belonging to the correct group, and in a case of binary classification this represents an outcome no better than random chance [108]. To avoid bias in the analysis, a cross-validation strategy should be adopted in the machine learning workflow [104]. Leave-one-out-cross-validation (LOOCV) is commonly used, especially in limited sample sets [14,24]. On a cautionary note, it is important to ensure the test data do not infiltrate the feature selection step prior to cross-validation, a common mistake in machine learning-based biomarker studies [109].

Many machine learning models also have a means of calculating the importance of each feature used to build the model. For example, decision tree-based algorithms report features with associated importance values, which correspond to their use in the decision tree and contribution to correct classification outcomes [105]. Other models assign variable importance of projection (VIP) scores to their features, and these values are used to identify which features are most useful in discriminating between unlike groups [12,13,14,16,61,62,64]. The method of extracting the useful features depends on the classification strategy used. The important features in the model can be identified as the lipids (or other molecules) most responsible for group distinction (i.e., biomarkers).

When a machine learning method is not used, ESI-MS sebum analysis is achieved via statistical tests such as the Spearman’s correlation test [4,5,19], Pearson’s correlation test [4,40], Student’s *t*-test [5,20,39,61,62,66,75], analysis of variance (ANOVA) [11,20,34,60,66], and/or the Mann–Whitney test [12,14,21,58,60,71]. While Student’s t-test and ANOVA evaluate the differences between group means, the Mann–Whitney test evaluates the differences in shape and medians between populations, and it is especially useful in the case of datasets lacking a normal distribution [110]. In these cases, it is necessary to perform a multiple comparisons test and therefore employ methods that control false discovery or family-wise error rate [111]. In ESI-MS sebum analysis, the Tukey test and Benjamini–Hochberg test are two common correction methods, though others have also been employed [5,20,34,45]. 

### 6.5. Lipid Identification 

Compound identification is an ongoing issue in the field of lipidomics and has been a topic of previous reviews [26,27]. Lipid identification relies on *m*/*z* mass accuracy and fragmentation pattern matching to lipidomic databases. However, definitive structure assignment is not always possible. Oftentimes, the mass accuracy results in multiple potential lipid matches, and the tandem MS fragmentation patterns may or may not deduce the exact structure of the lipid, especially in reference to the sn-position [26]. For example, because the exact structure of TGs often cannot be definitively assigned, there is a specific shorthand notation to signify this case: an underscore between fatty acid abbreviations (rather than a slash) denotes an unknown sn-position on the glycerol backbone [112]. Further developed lipid identification tools will be necessary as the field continues to progress.

## 7. Applications

### 7.1. Clinical Biomarkers

Sampling from the skin surface could lead to noninvasive testing for illness and disease [5,12,13,14,15,16,17,18,19,20,21,22], drug use [28,30,42,43,90,113], and dietary health [6,7], as well as new biomarker discovery for diseases currently considered difficult, if not impossible, to definitively diagnose [10,11]. This sampling would be especially advantageous for children and elderly patients, as it may help to better include these protected populations in future biomarker exploration studies in which they are currently underrepresented [23,24]. 

Several diseases are correlated with sebum lipids, and in some cases specific lipid biomarkers have been identified already. Cystic fibrosis, a disease that is often inaccurately diagnosed and requires an uncomfortable sweat-induced test, is linked to changes in sebum lipids, specifically diacylglycerol and fatty acid secretion [16,17]. Amid the COVID-19 global pandemic, researchers found sebum fatty acid amides and diacylglycerols to be correlated with the virus [13,15] as well as the overall decreased secretion of triglycerides and ceramides [14]. Parkinson’s disease is also linked to changes in high molecular weight lipids secreted on the skin [10,11]. Diabetes shows correlations with changes in lipid secretion [12]. Leprosy, of which testing currently requires a skin biopsy, causes changes in lipid secretion, specifically in mycolic fatty acids [22]. Sebaceous gland secretion tendencies are correlated with hair loss in instances of inflammatory disorders and chemotherapy treatments [44]. Atopic dermatitis is associated with changes in skin surface lipids, specifically those of ceramides, squalene, triglycerides, and propionic acid levels [5,18,19,20,21,44]. Disease diagnosis via skin surface sampling shows promise, but more research is needed to implement this strategy in the point-of-care setting. Since most sebum biomarker studies thus far are preliminary, further validation of these findings will be imperative to future therapeutic use. To accomplish this, additional, independent test datasets will be necessary to validate the accuracy of these earlier studies in large and diverse populations. Sampling from the skin would be advantageous, as it opens the possibility of new biomarker discovery for diseases currently lacking definitive biomarkers and it allows for noninvasive, painless clinical testing and monitoring.

### 7.2. Forensic Implications 

As mentioned previously, the sebum lipid profile is correlated with post-deposition time [8,9] as well as donor demographics [2,3,4,5,28,29]. This information could be crucial to eliminate suspects from a criminal investigation. For example, the magnitude of ozonolysis products may be indicative of whether or not a particular suspect’s fingerprint was deposited at the scene of the crime prior to the crime taking place [8,9]. Additionally, when only a partial fingerprint is available, an amount insufficient for traditional ridge-pattern identification, the lipidomic profile may help to narrow down the type of suspect, such as their age, sex, ethnicity, diet, and medications [3,7,42]. These analyses may also be useful in cases where there is a full fingerprint available at the crime scene, but the donor is not in the database, and therefore there are no potential matches. Though the deposition substrate and conditions are impossible to control when it comes to the scene of a crime, effective normalization strategies, and further research on the extent to which these parameters may affect the resulting lipid profile, could allow for significant advances in forensic-facing applications of ESI-MS sebum analysis.

## 8. Conclusions

Sebum lipid analysis via ESI-MS could lead to advances in noninvasive disease diagnosis as well as aid in proving one’s innocence (or lack thereof) in criminal investigations. Several studies correlate alterations in lipid profiles with differences in donor demographics, disease states, and time-since-deposition, but more research is needed to advance this methodology, and many of these findings have yet to be validated. The choice of sample collection and preparation protocol, as well as data acquisition and analysis methods, can either aid or hinder the production of meaningful, reproducible results. Ambient ESI-MS methods are highly efficient, but they are not the strategy of choice for unveiling the entire lipidomic profile of a sample. Lipid identification is an ongoing challenge, and the field would benefit from an expansion in this regard. Further research is needed to better understand and overcome the inherent biological and methodological variability of sebum lipid analysis before its widespread implementation in disease diagnostics and forensics. Both methodological considerations and post-acquisition normalization strategies should be advanced. Sebum lipid analysis is an intriguing field of study with great potential, and researchers can tap into this potential by developing an intentional workflow with the target application in mind.

## Figures and Tables

**Table 1:** Summary of previous approaches to sebum lipid collection, ESI-MS data acquisition, and subsequent data analysis.

Collection Substrate	Anatomical Location	Solvents	Separations and MS Analysis	Primary ESI-MS Lipid Analytes	Lipidomics Statistical Comparisons/Machine Learning	Purpose of Study	Reference
Glass slides	Fingerprints, forehead	N/A	DESI-LTQ Orbitrap (−)	FAs, TGs, DGs	GDBT, feature importance, cross-validation	Forensic applications	[3]
D-Squame	Forearm, forehead, cheek, chest abdomen, groin, thigh, heel, calf, shoulder, buttock, palm, hand, top of foot	MeOH, TCM, IPA	TriVersa NanoMate ion source, Q-Exactive (+/−)	CEs, DGs, TGs, CERs, Chol	Linear regression via least squares method, two-tailed Pearson and Spearman correlation, RF, cross-validation, PCA	Clinical applications	[4]
Sebutape	Cheeks	IPA, MeOH, water, toluene	Filtration, RP-LC-ESI-Qtrap (+/−)	CERs, FAs, oxylipins, nitrolipids,	Cauchy distribution, probabilistic PCA, full factorial MANOVA, Ward’s method for hierarchical clustering, Benjamini–Hochberg, Student’s *t*-test, Spearman’s rank correlation coefficient, Feltz and Miller test	Atopic dermatitis	[5]
Cotton swabs and phone surfaces (glass and plastic)	Hands	EtOH, water	RP-UPLC-ESI-QTOF (+)	FAs	Bray–Curtis dissimilarity analysis, RF, cross-validation, MSCluster, cosine scores, frequency histogram, heat mapping, 2D spatial molecular mapping	Forensic applications	[7]
Scintillation vials	Groomed fingerprints (forehead)	TCM	ESI-QTOF (−/+)	FAs	N/A	Forensic applications	[8]
Aluminum foil	Groomed fingerprints (forehead, nose, chin, and scalp)	TCM, water, IPA	RP-LC-ESI-QTOF (+)	TGs	In-house MIRION software package	Forensic applications	[9]
Cotton gauze	Back	MeOH, EtOH	Filtration, RP-LC- Synapt G2-Si QTOF (+)	CERs, TGs, DGs, MGs, SPs, FAs, STs	PLS-DA, AUC, ROC, bootstrapping, Pearson’s correlation matrix	Parkinson’s disease	[10]
Q-tip swabs	Back	N/A	IM-PSI (Synapt G2 Si) (+)	DGs, TGs, FAs, PLs	One-way ANOVA, probabilistic (Bayesian) method, RF, PCA, SVM	Parkinson’s disease	[11]
Sebutape	Forehead	TCM, MeOH, ACE, IPA	RP-UPLC-ESI-QTOF (+)	CERs, FAs, PLs, TGs, PRs	PLS-DA, PCA, VIP scores, heatmap analysis, Mann–Whitney U-test, ROC, AUC, topical overlap measurement	Type 2 diabetes	[12]
Silica plates	Back	MeOH, water	ESI-Q-Exactive Orbitrap (+)	FA amides, DGs, TGs, GPs, STs	Chi-squared independent test, Fisher’s exact test, Shapiro–Wilk test, Mann–Whitney hypothesis test, PLS-DA, VIP scores, permutation tests, *t*-test, volcano plots, linear SVM, ROC	COVID-19	[13]
Gauze swabs	Back	MeOH, EtOH	Filtration, RP-LC-ESI Orbitrap Q-Exactive Plus (+)	CERs, TGs, DGs	PLS-DA, LOOCV, VIP scores, two-tailed Mann–Whitney U-test, PCA, ROC, AUC, volcano plots	COVID-19	[14]
Gauze swabs	Back	MeOH, EtOH	RP-LC-ESI-Orbitrap Q-Exactive Plus (+)	TGs, DGs, CERs	PLS-DA, LOOCV, SVM, RF, K-nearest neighbors, Pearson correlation coefficient, VIP scores, PCA, logistic regression, recursive feature elimination with cross-validation, heatmapping	COVID-19	[15]
Silica plates	Back	MeOH, water	ESI-LTQ Oribtrap (−)	CERs, DGs, PLs	OPLS-DA, VIP scores	Cystic fibrosis	[16]
Glass slides	Forehead	N/A	DESI-LTQ Orbitrap (−)	FAs, DGs	GBDT, 6-fold rotational cross-validation, bootstrapping, ROC, AUC	Cystic fibrosis	[17]
Tape strips	Forearm	MeOH, TCM, n-hexane, IPA	SPE, NP-HPLC-ESI-Q	CERs	Bonferroni’s *post hoc* multiple comparison test	Atopic dermatitis	[18]
Sebutape	Back	MeOH, water, TCM, IPA	RP-LC-ESI-Q-Exactive (+/−)	TGs, MGs, DGs, PLs, CERs, FAs	PCA, OPLS-DA, R, Spearman’s correlation coefficient, *t*-test	Atopic dermatitis	[19]
D-Squame	Forearm	MeOH, water, TCM	RP-LC-ESI-Qtrap (+)	CERs, SMs	Student’s *t*-test, one-way ANOVA, Benjamini–Hochberg	Atopic dermatitis	[20]
D-Squame	Forearm	Water, MeOH, TCM	RP-LC-ESI-QTRAP (+)	CERs	Logistic regression analysis, chi-square test, Fisher’s exact test, Shapiro–Wilk test, Mann–Whitney U-test, ROC, Youden index method, Firth’s penalized method	Atopic dermatitis	[21]
Silica plates	Back	MeOH	ESI-LTQ Orbitrap (+)	PLs, CERs, FAs	PCA	Leprosy	[22]
Aluminum foil	Groomed fingerprints (cheek, neck, forehead)	DCM, MeOH, water	ESI-Orbitrap (+)	TGs, WEs	ROC, AUC, XGBoost, PCA, in-house R script for matrix building	Biomarker exploration	[24]
D-Squame	Forearm, chest, forehead	EtOH, MeOH, ACE, IPA	RR-RP-HPLC-ESI-QTOF/QqQ (−/+)	FAs, cholesterol sulfate, CERs	One-way ANOVA, Tukey’s multiple comparisons test, PCA, C-C plots, volcano plots, hierarchical clustering	Clinical applications	[34]
Sampling probes	Entire body	N/A	TD-ESI-LTQ (+/−)	SQ	Temperature color gradient	Clinical applications	[38]
Cyanoacrylate resin	Cheek, forearm, thigh, leg, back, palm	Hexane, EtOH, MeOH	Filtration, RP-HPLC-ESI-MS (+)	CERs	Student’s *t*-test, Mann–Whitney U-test	Skin sensitivity	[39]
Tape strips	Scalp, forehead, cheek, lip, arm, hand, palm, finger, buttock, leg	MeOH, TCM, n-hexane, IPA	SPE, NP-LC-ESI-Q (+)	CERs	Tukey’s test, Pearson’s correlation coefficient	Clinical applications	[40]
Tape strips	Forearm, cheek	MeOH, TCM, n-hexane, IPA	SPE, NP-HPLC-ESI-Q (+)	CERs	Student’s *t*-test, correlation coefficient	Clinical applications	[41]
Glass, paper, plastic, metal; followed by tape lifting	Groomed fingerprints (forehead)	N/A	DESI-LTQ Linear IonTrap (−)	FAs	N/A	Forensic applications	[42]
D-Squame	Cheek	MeOH	RP/HILIC-UPLC-ESI- Orbitrap Q-Exactive (+/−)	FAs, DGs, MGs, Chol, CERs, STs	Wilcoxon test, PCA, heat mapping, Benjamini–Hochberg, boxplots, volcano plots, random forest, VIMP scores, sparse Canonical Correlation Analysis, Pearson correlation coefficient, PLS regression, multiblock analysis, v-test	Environmental pollutant exposure	[45]
Tape strips	Forearm	MeOH, TCM, hexane, IPA	SPE, NP-LC-ESI-QqQtrap (+/−)	CERs	N/A	Clinical applications	[70]
N/A	Hand	N/A	SESI-API-QTOF (−)	FAs	N/A	Mosquito attractants	[56]
PDMS sampling patches	Axilla	N/A	TD-SESI-QTOF (−)	FAs	PCA	Clinical applications	[57]
Tape strips	Inner thighs and diaper-covered buttocks	MeOH, TCM, hexane, IPA	NP-LC-ESI-Q	CERs	N/A	Infantile skin	[59]
Tape strips	Cheek, buttock	MeOH, hexane, IPA, water, TCM	SPE, RP-UPLC-ESI-QqQ (+)	CERs, SMs	Mann –Whitney hypothesis test, one-way ANOVA, Tukey’s test, Spearman Rank, Pearson’s R correlations	Menopause	[60]
Sebutape	Cheek	TCM, MeOH, ACE, IPA	RP-UPLC-ESI-QTOF (+)	CERs, FAs, GLs, GPs, SPs, STs, PRs, SLs, PKs, SQ-related compounds, Chol, WEs	PCA, score plots, OPLS-DA, VIPs, Student’s *t*-test	Acne	[61]
Sebutape	Cheek	TCM, MEOH, ACE, IPA	RR-UPLC-ESI-QTOF (+)	FAs, GLs, GPs, SPs, STs, PRs, SLs, PKs	VIP, *t*-test, PCA score plots, OPLS-DA score plots	Blue light therapy acne treatment	[62]
Glass slides	Forehead	TCM, MeOH, IPA, Methyl-tert-butyl ether	RP-LC-Nano-ESI- Q-Exactive (+/−)	TGs, CEs, CERs DGs, Chol, PLs, FAs	Heat mapping, Venn diagrams	Inflammation	[63]
Sebutape	Cheek	TCM, MeOH, ACE, IPA	RP-UPLC-ESI-QTOF (+)	CERs, FAs, GPs, TGs, DGs,	PLS-DA score plots, S-plots, VIP scores	Skin sensitivity	[64]
Tape strips	Forearm	MeOH, TCM	RP-UPLC-ESI-QqQ (+/−)	CERs	Student’s *t*-test	Clinical applications	[65]
Sebutape	Forehead	EtOH, butylated hydroxytoluene, ethyl acetate, MeOH, Me_2_O, IPA	RR-RP-HPLC-ESI-TOF (+/−)	TGs, DGs, MGs, FAs, WEs, PRs, triterpenes, SLs, STs	Mass Profiler Professional volcano plots, in-house statistical method, Bonferroni’s correction, PLS, PLS weights plot, Student’s *t*-test, ANOVA, post hoc Tukey’s honestly significant difference test	Acne	[66]
Sebutape	Forehead	EtOH, ethyl acetate, ACE, MeOH, IPA	RR-RP-HPLC-ESI-TOF/QqQ (+/−)	FAs, TGs, DGs, CEs, WEs, SQ	N/A	Understanding the sebum lipidome	[67]
D-Squame	Forearm	Cyclohexane, EtOH, TCM, MeOH	Silica acid column chromatography-nanoESI-LTQ (−)	CERs	N/A	Clinical applications	[68]
Tape strips	Forearm	MeOH, IPA, TCM, hexane	SPE, RP-LC-ESI-QTOF (+/−)	CERs	N/A	Clinical applications	[69]
D-Squame	Forearm	Water, MeOH, TCM, ACE, ACN	RP-LC-ESI-QqQ (+)	Derivatized FAs	Shapiro–Wilk normality test, two-tailed Mann–Whitney test	Atopic dermatitis	[71]
Cyanoacrylate resin and Teflon extraction funnel	Forearm	ACE, EtOH, TCM, MeOH, hexane, EtOH	RP-LC-ESI-Ion Trap (−)	CERs	N/A	Clinical applications	[72]
Cyanoacrylate resin	Forearm	Hexane, EtOH, diethylether, MeOH	RP-LC-ESI-Ion Trap (−)	CERs	N/A	Clinical applications	[73]
D-Squame	Forearm	Water, MeOH, TCM	RP-LC-ESI-Qtrap (+)	CERs	Benjamini–Hochberg, Tukey’s type boxplots, random forest, Gini index	Dupilumab therapy for atopic dermatitis	[74]
Leukoflex tape strips	Cheek	Ethyl acetate, IPA, water, MeOH	Filtration, RP-UPLC-ESI-QqQ (+)	CERs	Correlation coefficients, Cook’s score, unpaired *t*-tests corrected for multiple comparisons	Acne	[75]
N/A	Hand	N/A	SESI-Q-Exactive Orbitrap (+)	SQ	Clustergram hierarchical cluster analysis, OPLS-DA, VIP scores, S-plot	Air quality	[76]
Scraping method	Foot sole	TCM, MeOH	RP-HPLC-ESI-ion trap (−)	CERs	N/A	Clinical applications	[77]
N/A	Finger	EtOH, water	DESI-LTQ Orbitrap/LCQ Duo Quadrupole Ion Trap (+/−)	FAs	N/A	Direct skin analysis	[78]
Filter slip	cheek	MeOH	zvPSI-Orbitrap (+/−)	FAs, TGs, DGs, MGs, WEs, CEs, CERs, SMs, GPs	N/A	Voltage-free analysis of diverse samples	[79]

## Abbreviations

ACE	acetone
ANOVA	analysis of variance
AUC	area under the ROC curve
CE	cholesterol ester
CER	ceramide
Chol	cholesterol
DCM	dichloromethane
DESI	desorption electrospray ionization
DG	diglyceride
ESI	electrospray ionization
EtOH	ethanol
FA	fatty acid
GDBT	gradient boosting tree ensemble
GL	glycerolipid
GP	glycerophospholipid
HILIC	hydrophilic interaction liquid chromatography
HPLC	High-performance liquid chromatography
IM	ion mobility
IPA	isopropanol
LC	liquid chromatography
LOOCV	leave-one-out cross-validation
MeOH	methanol
MG	monoglyceride
NP	normal-phase
OPLS-DA	orthogonal partial least squares discriminant analysis
PCA	principal component analysis
PK	polyketide
PL	phospholipid
PLS-DA	partial least squares discriminant analysis
PR	prenol lipid
PSI	paper spray ionization
Q	single quadrupole
QqQ	triple quadrupole
RF	random forest
ROC	receiver operator characteristic
RP	reversed-phase
RR	rapid resolution
SESI	secondary electrospray ionization
SL	saccharolipid
SM	sphingomyelin
SP	sphingolipid
SPE	solid-phase extraction
SQ	squalene
ST	sterol lipid
SVM	support vector machine
TD	thermal desorption
TG	triglyceride
TOF	time-of-flight
TCM	trichloromethane (chloroform)
UPLC	ultra performance liquid chromatography
VIP	variable importance of projection
WE	wax ester
XGBoost	extreme gradient boosting
zvPSI	zero-volt paper spray ionization
